# Photodynamic Antimicrobial Chemotherapy for Root Canal System Asepsis: A Narrative Literature Review

**DOI:** 10.1155/2015/269205

**Published:** 2015-12-10

**Authors:** P. Diogo, T. Gonçalves, P. Palma, J. M. Santos

**Affiliations:** ^1^Faculty of Medicine, University of Coimbra (FMUC), Avenida Bissaya Barreto, 3000-075 Coimbra, Portugal; ^2^Centre for Neuroscience and Cell Biology (CNC), University of Coimbra, Coimbra, Portugal

## Abstract

*Aim.* The aim of this comprehensive literature review was to address the question: Does photodynamic therapy (PDT) improve root canal disinfection through significant bacterial reduction in the root canal system?* Methodology.* A comprehensive narrative literature review was performed to compare PDT effect with sodium hypochlorite as the comparative classical irrigant. Two reviewers independently conducted literature searches using a combination of medical subject heading terms and key words to identify relevant studies comparing information found in 7 electronic databases from January 2000 to May 2015. A manual search was performed on bibliography of articles collected on electronic databases. Authors were contacted to ask for references of more research not detected on the prior electronic and manual searches.* Results.* The literature search provided 62 titles and abstracts, from which 29 studies were related directly to the search theme. Considering all publications, 14 (48%) showed PDT to be more efficient in antimicrobial outcome than NaOCl (0.5–6% concentration) used alone and 2 (7%) revealed similar effects between them. Toluidine blue and methylene blue are the most used photosensitizers and most commonly laser has 660 nm of wavelength with a 400 nm diameter of intracanal fiber.* Conclusions.* PDT has been used without a well-defined protocol and still remains at an experimental stage waiting for further optimization. The level of evidence available in clinical studies to answer this question is low and at high risk of bias.

## 1. Introduction

The main goal of endodontic treatment is to prevent and, when required, to cure apical periodontitis and maintain or reestablish periapical tissue health [1]. To accomplish this objective, it is mandatory to control the microbial load inside the root canal system. The chances of a favourable outcome with endodontic treatment are significantly higher if infection is eradicated effectively by chemomechanical preparation before the root canal is obturated. However, if positive cultures can be obtained from the root canal at the time of root filling, there is a higher risk of treatment failure [2]. In an attempt to improve disinfection, an interappointment dressing has been advocated to diminish the percentage of root canals with no cultivable microorganisms in comparison to those only treated with chemomechanical preparation. Nevertheless, the two-visit treatment protocol did not improve the overall antimicrobial efficacy of the treatment [3]. Indeed, in all cases where viable microorganisms remain in the root canal system, the prognosis for repair is adversely affected [2, 3].

Presence of a* smear layer* after instrumentation reduces effectiveness of irrigants and temporary dressings in disinfecting dentinal tubules. Moreover, complexity of anatomy translated into root canal system with its isthmuses, ramifications, and fins [4] turns complete elimination of bacteria using instrumentation and irrigation into an almost impossible task. Besides, bacteria persisting in biofilms show diverse phenotypes when compared with planktonic cells, including increased resistance to antimicrobial agents [5]. It has been assessed that bacteria in biofilms are approximately 1000-fold less susceptible to effects of commonly used antimicrobial agents than their planktonic equivalents and are highly unaffected with phagocytosis by immune system [6]. There are several mechanisms used by bacteria which allow them to adapt to the environment [7]. Biofilm formation [8], stress response [9], physiological adaptation [7], and the beginning of subpopulations of cells are among some of the adaptive mechanisms used by bacteria along with various systems involving the exchange of genetic material [10] between bacteria. These mechanisms can support bacterial survival under the limiting environments, such as that found in the root canal. One of the most relevant features of adaptation for oral bacteria is the adhesion to surfaces leading to the formation of plaque biofilms, which not only serves to aid in their retention but also results in increased survival rate [11]. Biofilms form when planktonic bacteria in a natural liquid phase are deposited on a surface containing an organic conditioning polymeric matrix or conditioning film [7]. In this dynamic process, several organisms coadhere to the surface [12] and grow with certain cells detaching from the biofilm over time. Biofilm formation in root canals, as postulated by Svensater and Bergenholtz [13], is probably initiated at the moment of the first invasion of the pulp chamber by planktonic oral organisms after some tissue breakdown.

Biofilm disruption and disinfection of root canals are the most critical steps during treatment of an infected root canal system, which are essential to avoid persistence of microbial infection and achieve endodontic success [14]. The mode of action and efficacy of a wide variety of cleaning, antimicrobial, and disinfecting agents such as NaOCl, chlorhexidine, ethylenediamine tetraacetic acid (EDTA), citric acid, hydrogen peroxide, halogens, and ozone have been investigated [15–18]. Disinfecting agents and antimicrobial medicinal products routinely used in endodontics can be inactivated by dentin, tissue fluids, and organic matter [6, 19]. Moreover, some microbial species, such as* Enterococcus faecalis* [20, 21] and* Candida albicans* [22, 23], show resistance to those agents and their efficacy is dependent on the concentration achieved and time of contact [24]. Most of these disinfectants with effective bactericidal activity are used at subtoxic level, but also at concentrations where toxicity is becoming a significant factor. Searching for new methods to provide extra disinfection for root canal system without cytotoxic effects and to improve treatment outcome, innovative techniques including various laser wavelengths [25], hydraulic [26], sonic, and ultrasonic irrigation [27–29], nanoparticles [30], inactivation of efflux pumps [31], and photodynamic therapy (PDT) has been proposed in literature.

PDT was discovered by chance at the very beginning of the twentieth century, when a combination of nontoxic dyes exposed to visible light resulted in microorganism cell death. As reviewed by Henderson and Dougherty in 1992 [32], Oscar Raab, a medical student working with Professor Herman Von Tappeiner in Munich, introduced the concept of microbial cell death induced by interaction of light and chemicals [32]. During the course of Raab's study, he demonstrated that the combination of light and dyes was much more effective in killing the microorganism* Paramecium*.

Those observations were repeated with a diversity of uni- and multicellular organisms. Succeeding work in this laboratory coined the term* photodynamic action* and demonstrated presence of oxygen as an essential requisite for photosensitization to occur. Years later, Dougherty and coworkers clinically tested PDT in cutaneous/subcutaneous malignant tumours. However, it was John Toth who renamed this therapy as PDT. Combined effect of three elements,* light*,* PS*, and* oxygen*, has been termed* photodynamic antimicrobial chemotherapy* by Wainwright [33] and also recognized as* antimicrobial photodynamic therapy* [34] and* photoactivated disinfection* [35].

PDT uses a nontoxic dye, known as photosensitizer (PS), on a target tissue, which is consequently irradiated with a suitable visible light of the appropriate wavelength to excite the PS molecule to the singlet state in presence of oxygen to produce reactive oxygen species (ROS) [36]. When PS absorbs light, this excited state may then undergo intersystem crossing to the slightly lower energy, but the longer lived, triple state can undergo two kinds of pathways known as Type I (reacting with the substrate) and Type II (reacting with molecular oxygen) photoprocesses. Both pathways require oxygen.

The* type 1 radical and reactive oxygen species* pathway comprises an* electron transfer step* between the triplet PS and a substrate with generation of radical species. The finalist is then intercepted by ground state molecular oxygen yielding a variety of oxidized products. The baseline PS has two electrons in opposite spins (singlet state) in the low energy molecular orbital. Subsequent to the absorption of light, one of these electrons is boosted into a high-energy orbital but keeps its spin (first excited singlet state). This is a short-lived time species, nanoseconds, and can lose its energy by emitting light (fluorescence) or by internal conversion into heat. Type 1 pathway frequently involves initial production of superoxide anion by electron transfer from the triplet PS to molecular oxygen (monovalent reduction) initiating radical-induced damage in biomolecules. Superoxide is not particularly reactive in biological systems and does not by itself cause much oxidative damage but can react with itself to produce hydrogen peroxide and oxygen, a reaction known as* dismutation* that can be catalyzed by the enzyme superoxide dismutase (SOD). The way of the electron relocation between the PS and the substrate is controlled by the relative redox potentials of the two species.

*Type 2 pathway*,* singlet oxygen*, involves an electronic* energy transfer process* from the triplet PS to a receptor, most frequently oxygen, which is a triplet in its ground state. The final compound is converted to a highly reactive species, the singlet oxygen (^1^O_2_). The excited singlet state PS may also undergo the process known as* intersystem crossing* whereby the spin of the excited electron inverts to form the relatively long-lived, in terms of microseconds, excited triplet state that has parallel electron spins. The long lifetime of the PS triplet state is explained by the fact that the loss of energy by emission of light (phosphorescence) is a* spin forbidden* process, as the PS would move directly from a triplet to a singlet state. Photosensitized processes of types 1 and 2 depend on the initial involvement of radical intermediates that are subsequently scavenged by oxygen or the generation of the highly cytotoxic singlet oxygen (^1^O_2_) by energy transfer from the photoexcited sensitizer. It is difficult to determine without doubt which of these two mechanisms is more prevalent; both types of reactions can happen simultaneously and the ratio between them depends on three singular features: oxygen, substrate concentration, and PS type [37].

Hamblin and Hasan in 2004 [36] stated that antimicrobial PS can be divided into three categories: (I) those that strongly bind and penetrate the microorganisms (chlorin e6), (II) those that bind weakly as toluidine blue (TB) and methylene blue (MB), and (III) those that do not demonstrate binding at all such as rose bengal (RB). Understanding these mechanisms of action is essential because, in bacterial cells, outer membrane damage plays an imperative role, differently from mammalian cells, where the main targets for PDT are lysosomes, mitochondria, and plasma membranes [38]. Typically, neutral anionic or cationic PS molecules could powerfully destroy Gram-positive bacteria, whereas only cationic PS or strategies which attack the Gram-negative permeability barrier in combination with noncationic PS are able to kill multiple logs of Gram-negative species [39]. This difference in susceptibility between species in the two bacterial types is explained by their cell wall physiology. To understand better the PDT effect in those microorganisms, it is very important to analyse in detail the microbial cell walls. In* Gram-positive bacteria*, the cytoplasmic membrane is surround by a relatively porous peptidoglycan layer and lipoteichoic acid that allows the PS to cross. Different from this, the* Gram-negative bacteria* cell envelope consists of an inner and an outer membrane which are separated by a peptidoglycan layer. The outer membrane forms an effective permeability barrier between the cell and the environment and tends to restrict the binding and penetration of several PS. Fungi are provided with a thick cell wall that includes beta glucan and chitin offering a permeability barrier. In terms of PDT efficacy, in fungal wall, it was described as having an intermediate behavior between Gram-positive and Gram-negative bacteria [40]. On the basis of these considerations, it appears that Gram-negative bacteria represent the most challenging targets for any form of antimicrobial treatment. The mechanism of action of basic polymer PS conjugates is thought to be that of* self-promoted uptake pathway* [41]. In this method, cationic molecules first dislocate the divalent cations, such as calcium (Ca^2+^) and magnesium (Mg^2+^), from their position on the outer membrane where they act as an anchor for the negatively charged lipopolysaccharides molecules [40, 41]. The debilitated outer membrane becomes slightly more permeable and allows even more of the cationic PS to gain access thus steadily increasing the disorganizations of the permeability barrier increasing PS uptake with each additional binding. It is thought that host cells only gradually take up cationic molecules by the process of endocytosis, while their uptake into bacteria is relatively fast [39]. Further important observation that has been made about these cationic antimicrobial PS concerns their selectivity for microbial cells compared to host mammalian cells [37]. These findings are relevant, because photoaction occurs in direct contact with membranes [42]. The PS efficiency in generating ROS within membranes is dependent on the intrinsic characteristics of the PS in aqueous solution as well as their partition in the membrane [42]. The early attack of singlet oxygen in membranes lipids is by the specific reaction with double bonds to form allylic hydroperoxides; the efficiency of this reaction is dependent on the lowest ionization potential of the olefins and also on the availability of allylic hydrogens [42].* Photodynamic lipid peroxidation* is an oxidative degradation of cell membrane lipids, also known as* photoperoxidation*, and it has been related to several microbial cytotoxic effects, such as increased ion permeability, fluidity loss, inactivation of membrane proteins, and cross-linking, which disrupts the intracellular homeostasis. Consequently, necrosis is induced as a cell death process. A probable explanation is that PS bound to the membrane and generates most of the singlet oxygen, ^1^O_2_, involved in photoperoxidation [43] highlighting the double selectivity (light and PS cellular localization) and the fact that it works in multiresistant strains and does not encourage resistance [42]. PDT's lethal action is based on photochemical production of ROS and not thermal and cavitation effects, as is the case with high power laser therapy [44]. One of several PDT's advantages clinically is the absence of thermal side effects in periradicular tissues [45] and this property of PDT aspect makes it highly effective in eradicating microorganisms such as bacteria [45], viruses [46], and fungi [47] without causing damage of adjacent tissues due to overheating [45].

In recent years, PDT has been applied in several areas, particularly in periodontology [48–50], in general dentistry [51] and also in endodontic field as an adjunct of classical irrigation solutions in root canal disinfection [52, 53]. These studies suggest PDT's potential as a therapeutic weapon, which aims to support endodontic antimicrobial treatment, especially enhancing irrigation solutions effect. The purpose of this narrative comprehensive literature review is to answer the focused question, “*Is antimicrobial PDT efficacy better than that of sodium hypochlorite's in root canal treatment?*” For this analysis of the literature, we selected and analysed 29 studies using antimicrobial PDT in endodontic field, highlighting methodologies used and their reported effectiveness and efficacy.

## 2. Materials and Methods

### 2.1. Criteria in Selection of Studies

For this comprehensive narrative literature review [54], eligibility criteria were (I) articles published in English language; (II) original papers; (III) experimental studies (*in vitro* and* ex vivo*); (IV) clinical studies (*in vivo*); and finally (V) scientific reports of PDT efficacy in root canal disinfection/asepsis. The exclusion criteria were (I) unpublished data, (II) conference papers, (III) historic reviews, (IV) letters to editor, and (V) papers due to PDT outcomes in other fields (outside of endodontics).

**Figure 1:**
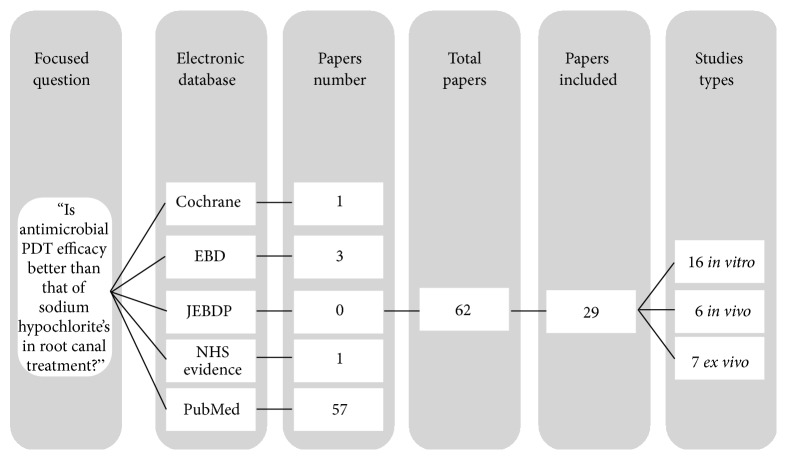
Identification of studies used in this narrative review.

As a first step, the aim was to investigate the terms “Endodontic”, “Photodynamic Therapy”, and “Antimicrobial Disinfection”. Briefly, we used PubMed to identify Medical Subject Headings (MeSH) terms corresponding to each term. Nevertheless, MeSH terms use is not common to all articles, making this search method infeasible. Then, exhaustive automated searches of Cochrane Collaboration, Evidence Based Dentistry (EBD), Journal of Evidence-Based Dental Practice (JEBDP), NHS Evidence, and PubMed (Figure 1) were performed from January 2000 up to and including May 2015 using various combinations of the following key indexing terms: (a)* endodontic photodynamic therapy*; (b)* antimicrobial photodynamic therapy*; (c)* photo-activated disinfection*; (d)* light-activated disinfection*; (e)* laser-assisted photosensitization*; (f)* root canal disinfection*; and (g)* endodontic lasers*.

Titles and abstracts of all articles resulting from electronic search were screened independently and in duplicate by 2 reviewers. The review itself was performed to reject articles that did not meet inclusion criteria. Any disagreement between reviewers was solved via debate, although in specific cases of disagreement that were not resolved with discussion, opinion of a senior commentator was required. Hand searching of reference lists of original and reviewed articles that were found to be relevant was also performed.

In a second step, full-text copies of all remaining articles were obtained and further meticulous assessment was performed independently by each reviewer to determine whether or not they were eligible for this study based on the specific inclusion and exclusion criteria cited above and proven for agreement.

Quality evaluation of randomized clinical trials and observational studies was performed using STROBE [55] (strengthening the reporting of observational studies in epidemiology) and CONSORT [56] (consolidated standards of reporting trials) statement criteria, respectively.

## 3. Results

### 3.1. PDT Antimicrobial Efficacy in Included Studies

Literature search provided 62 titles and abstracts; from those, 29 studies concerned this theme: 16 were performed in* in vitro* conditions, 6 were* in vivo* studies, and the last 7 readings were* ex vivo*. From all 29 papers included in this review, 16 (55.2%) were* in vitro* studies (Table 1).

In data processing, authors classified all studies in three categories:* category I*,* in vitro*;* category II*,* in vivo*; and finally,* category III*,* ex vivo*, to describe and clarify studies' details. In category I, 16* in vitro* studies, only 5 (31%) [57–61] reveal best antimicrobial PDT outcomes when compared with sodium hypochlorite (NaOCl) in range of 0.5 to 6%. Only one study performed by Nagayoshi et al. [62] reveals equal results between PDT and NaOCl; the remaining 10 (62.5%) studies [63–72] showed PDT outcomes unhelpful when compared with NaOCl as a classical irrigant solution, in concentration range of 0.5 to 6%. In category II, 6 (21%) papers [35, 58, 73–76] were analysed (Table 2).

All were performed in the human dentition, five [35, 58, 73, 74, 76] were performed in permanent dentition, and only one was achieved in deciduous teeth by Prabhakar et al. [75]. All studies in category II (100%) presented that PDT efficacy overthrew (0.5–2.5%) NaOCl. Considering tooth type and its influence in PDT efficacy outcomes, Garcez et al. group [58, 74] and Jurič et al. [76] tested only permanent uniradicular human teeth (incisors and canines) as samples. However, Prabhakar et al. [75] considered deciduous molars as a prerequisite for his study. Finally, Bonsor et al. [35, 73] used not only uniradicular but also permanent multiradicular teeth. In terms of endodontic diagnosis, Garcez et al. [58] in his first study used patients with necrotic pulps and periapical lesion; then, in 2010, his group [74] performed a second study to assess PDT efficacy in teeth with previous endodontic treatment, endodontic retreatment. Jurič et al. [76] in 2014 evaluated PDT antimicrobial outcomes efficacy applied also in endodontic retreatment. Both studies [74, 76] revealed PDT outcomes near 100% effective.

In category III (*ex vivo*), 7 (24%) papers [5, 77–82] were analysed (Table 3).

Based on this, 3 (43%) studies [5, 78, 79] revealed superior PDT outcomes compared to 0.5–6% of NaOCl and in one study by Xhevdet et al. group [81] showed 2.5% NaOCl irrigation showed similar results to 5 min irradiation of PDT, 10 mg mL^−1^ phenothiazine chloride as PS irradiated with 660 nm light source.

Considering all 29 publications, 14 of them (48%) [5, 34, 35, 57–61, 73–76, 78, 79] showed best PDT antimicrobial outcome compared to (0.5–6%) NaOCl used alone; 2 (7%) [62, 81] papers reveal similar effects between them and the last 13 (45%) [63–72, 77, 80, 82] studies revealed supremacy of sodium hypochlorite (0.5–6%).

### 3.2. Antimicrobial PDT Outcomes

The present narrative literature review was based on hypothesis that antimicrobial PDT efficacy was better than sodium hypochlorite in root canal asepsis. Considering all studies chronologically organized in Table 4, 48% (14 papers) showed PDT is more efficient than NaOCl (0.5–6% concentration) used alone and 7% (2 papers) reveal similarity in antimicrobial outcome effects between them.

On the other hand, 45% (13 studies) of studies reveal supremacy of sodium hypochlorite. From all studies, it must be observed that 55.2% (16 studies) were conducted at* in vitro* conditions, revealing preferential experimental phase where PDT remains in the last two decades. This must be taken into consideration, when comparing with clinical PDT studies, in which evidence reveals unanimous evidence supremacy of PDT over NaOCl.

### 3.3. Evaluation Parameters

The 29 studies analysed for this review revealed assessment of antimicrobial PDT efficacy was done through several parameters, from microbiological evaluation (classical analysis) to recent advanced imaging approaches. At the beginning, bacteriological experimental* in vitro* studies presented results through colony-forming units (CFU). This approach overcomes limitation of direct microscopic counting of bacterial cells, where all cells, dead and live, are counted; CFU estimates only viable cells of each group, before and after treatment, in planktonic suspensions and biofilms. Results are given as CFU/mL (colony-forming units per millilitre) for liquids. This approach was used in 24 studies (83%) [34, 57–66, 68–72, 74–81]; Bonsor et al. [35, 73] used bacterial load scores, instead of the usual CFU, to evaluate PDT antimicrobial efficacy in clinical studies. Muhammad et al. [82] in 2014 over an* ex vivo* study elected the same evaluation unit as in Bonsor et al. studies, repeating bacterial score, complemented with microbiological identification.

Scanning electron microscopic (SEM)* in vitro* investigations have demonstrated the penetration of bacteria up to 1000 *μ*m into dentinal tubules and hence it is very difficult for normal irrigants to penetrate till this depth. NaOCl can penetrate in a range of 60–150 *μ*m into dentinal tubules and of Nd:YAG laser at a range of 400–850 *μ*m.* Enterococcus faecalis* is known to colonize dentinal tubules up to depth of 600–1000 *μ*m and conventional irrigants cannot penetrate more than 100 *μ*m [83]. With SEM, Bumb et al.'s [61]* in vitro* study revealed bacteria found till the depth of 980 *μ*m (control group) and in PDT group achieved a depth of 890–900 *μ*m free from microorganisms, which revealed PDT as a promising root canal disinfection approach. SEM is a remarkably versatile technique, which reproduces the exact morphology of structures, but as the main disadvantage of dehydration of the sample. It was used in 10 (34%) studies [61, 65, 68, 69, 71, 72, 79–82] and ESEM (environmental scanning electron microscope) [84] which allows preservation of the sample before and after light irradiation was not used in any study. CSLM was used only in one study of George and Kishen [59] showing capability of obtaining in-focus images from selected depths allowing three-dimensional reconstruction of topologically complex objects with a specific hardware analysis. The same study [59] also evaluated dark toxicity (detail described in photosensitizers subchapter) and ROS production. PDT antimicrobial killing can be mediated by type I and type II reactions, although singlet oxygen is the predominant chemical entity causing cell death. Analysis and quantification of singlet/reactive oxygen species detection seem to be an excellent methodology to quantify antimicrobial PDT outcomes. However, of all studies analysed, only George and Kishen [59] performed ROS quantification and state that the increased photooxidation potential and singlet oxygen generation were thought to have collectively contributed towards the biofilm matrix disruption [59] and bacterial inactivation.

### 3.4. Photosensitizers

Photosensitizers (PS), which were preferentially located at the bacterial cytoplasmic membrane, have been found to be very potent photoantimicrobial agents. One important exception is represented by acridines [36], such as proflavine or acridine orange, which mostly interpolate with DNA bases. Highest modifications of cell functions and morphology, triggered by photodynamic inactivation, are typically due to damaged membranous domains [36]. This pattern of photoinduced subcellular damage is in agreement with lack of mutagenic effects [85], as well as with absence of selection of photoresistant microbial strains even after several photosensitization treatments.

Methylene blue (MB), a well-established PS, has been used in PDT for targeting endodontic bacteria since 2007 [34] and remains as one of the most used; but the first PS used in endodontic field was toluidine blue (TBO) [63]. Hydrophilicity of MB, along with its low molecular weight and positive charge, allows it to cross outer membrane of Gram-negative bacteria through porin channels [33, 86]. MB predominantly interacts with anionic macromolecule lipopolysaccharide, resulting in generation of MB dimers, which participate in the photosensitization process. From all studies evaluated, 12 (41%) [35, 63–65, 67, 68, 70, 71, 73, 79, 80, 82] used TBO as PS, while 10 (34%) [5, 59, 61, 65, 66, 69, 75, 77, 78, 87] studies used MB. One study, elaborated by Souza et al. [65], used both TBO and MB as PS. The best antimicrobial PDT results were achieved with TBO and MB as PS in the same concentration, 15 *μ*g mL^−1^ [5, 65, 70, 71, 82]. All concentration variations are studied first in preliminary findings to obtain fluorescence characteristics [45] in ultraviolet-visible absorption spectra on a diode-array spectrophotometer to understand absorption pattern and to establish final concentration. In designing criteria for definition of second generation PS, an essential feature has been evaluated,* dark toxicity* [88]. It is clearly desirable that PS has zero or very low cytotoxicity in total absence of light and this indicates antimicrobial PDT efficacy results strictly from combination between PS and light source. Reviewing literature in this aspect, only one study from George and Kishen [59] had this aspect in mind.

The period of intimate contact between PS and substrate without irradiation, known as preincubation time (PIT), diverges in terms of PS used. It is also important that PIT is fixed in total absence of light, even natural light [88]. The most used TBO PIT was 60 seconds (s) [35, 67, 73, 80, 82] from a range of 30–300 s (mean = 95.5 s) and MB PIT most used was 300 s [5, 66, 75, 78] from a range of 60–600 s (mean = 353.3 s).

### 3.5. Light

Phototherapy describes use of light in treatment of disease; photochemotherapy, on the other hand, involves a combination of administration of a photosensitizing agent followed by action of light on tissues in which the agent is located [89]. PDT kills microorganisms by combined action of visible light and a photosensitizing dye. From all 29 studies evaluated, laser wavelength gap referred to in literature was between 380 [60] and 910 nm [61] (mean = 650.8 nm), while most used light source was a diode laser of 660 nm [5, 34, 58, 65, 66, 69, 74–77, 79, 81] wavelength. Some orthodox photosensitizers have lost their proficiency because they needed specific light source for each one and combination between them triggers the costs. Several examples can illustrate this aspect: Azpaste (685 nm) [57]; indocyanine green (805 nm) [62]; eosin-Y, and curcumin (380–500 nm) [60] which make them, nowadays, outdated.

In terms of commercial light sources, there are three diode lasers that authors would like to remark:* Denfotex* of 635 nm (SaveDent; Denfotex, Inverkeithing, UK) [64, 90, 91],* Helbo* of 660 nm (Helbo Photodynamic Systems, Grieskirchen, Austria) [91], and* FotoSan* emitting in the red spectrum with a power peak at 628 nm (FotoSan; CMS Dental, Copenhagen, Denmark) [67, 68, 71]. Delivery of PDT treatment with Denfotex, according to the manufacturer's recommendations, includes TBO as PS at a concentration of 12.7 mg L^−1^, applied in 120 s as preincubation time (PIT); followed by an irradiation time (IT) of 150 s with a laser output power of 100 mW using the spherical tip. Helbo system advocates Helbo Endo Blue PS, a MB dye, at a concentration of 10 mg L^−1^ fully covering the root canal with a PIT of 180 s; after this time, according to the manufacturer's recommendations, excess PS dye should be removed and light source applied for an IT of 120 s and an output power of 75 mW with an attached 2D spot probe Helbo Photodynamic Systems. Meire et al. in 2012 [91] performed an* in vitro* study comparing Denfotex with Helbo. The same team [91] reported that log reduction with Helbo system was higher than with Denfotex; however, the best results were achieved with 2.5% NaOCl for 300 s. Several differences between the two systems were described and might account for the distinctive reduction outcomes in viable cells [91]. First, the PS dyes are chemically different; secondly, Helbo Blue PS is much more concentrated than Denfotex PS. Thirdly, following the PS application and the recommended PIT, the PS excess has to be removed with the Helbo system, dried canal [91], but not with the two other systems: Denfotex and FotoSan, where fiber is inserted in the liquid [67, 68, 71, 91]. In the three PDT systems, all probes are different. While the Helbo systems 2D spot probe is designed for two-dimensional exposure, Denfotex and FotoSan tips emit in three dimensions and this has strong implications for energy densities at the target. Also the lasers wavelengths are slightly different. It seems that there is also a clear reduction in light exposure as irradiation time (IT): Denfotex (150 s) [91], Helbo (120 s) [91], and FotoSan (30 s) [67, 68, 71].

FotoSan uses only TBO as a FotoSan PS, available in three types of viscosities (low, medium, and high), all at the same concentration (100 *μ*g mL^−1^) and the light source with an output power of 100 mW. FotoSan was evaluated in 3 (10.3%) studies [67, 68, 71], curiously all conducted in* in vitro* conditions with FotoSan protocol IT of 30 s.

Poggio et al. [67] tested 30 s and also 90 s of IT and declared that with the longer light exposure, it results in an increased percentage of bacterial reduction for different groups of* Enterococcus faecalis*,* Streptococcus mutans*, and* Streptococcus sanguis* strains. For this reason, this group admits that FotoSan needs to be applied into canal for at least 90 s, because 30 s of irradiation showed lower performance when compared to PDT with IT of 90 s, although the same group reveals that the best outcomes were achieved with PDT 30 s of IT combined with 5% NaOCl.

Irradiation time (IT) is an important issue to considerer and, in this parameter, PDT studies outcomes are very dissimilar with a range between 30 s [63, 68] and 1800 s [60]. Considering the most used wavelength of 660 nm, preference irradiation time is in the range between 30 s [5] and 1200 s [77] (mean = 223 s).

The last aspect considered in laser literature is the need for an intracanal fiber tip to spread light into root dentinal walls as well as within biofilms. From all studies analysed, only Nunes et al. [66] explored* in vitro* effectiveness of PDT with and without use of an intracanal optical fiber. Nunes et al. [66] concluded that, under experimental conditions, PDT was effective against* E. faecalis*, regardless of whether or not it is applied through an intracanal fiber. Considering the use of intracanal fiber, only 4 (13.8%) studies [63, 70, 75, 80] were not performed with intracanal fiber (Table 5).

Prabhakar et al. [75], in these particular conditions, revealed in a clinical study that antimicrobial PDT performance is better than 0.5% NaOCl. When PDT is implemented in planktonic suspensions established in multiwells, light source was applied 20 mm [60] away from well. Considering intracanal fiber, fiber tip diameter most used was 400 nm [59, 62, 64, 72, 77]. In terms of intracanal fiber location inside root, it varies from full working length (WL) [34, 58, 62, 64, 66, 71, 76, 79, 81, 82], the most prevalent, to WL-1 millimeters (mm) [57, 74], WL-2 mm [61, 68], WL-3 mm [67–69], and WL-4 mm [35, 73]. Contemplating the same device, intracanal fiber, in terms of applying movements to itself or inserting endodontic tip static inside root canal to improve the best light diffusion through root canal [66]. The former was applied in 5 studies [34, 58, 65, 66, 79] with spiral movements from apical to cervical and latter maintained static [64, 76, 77] inside root canal orifice [77] or at WL [64, 76].

### 3.6. Disinfection Protocol

In literature, when PDT studies are accomplished in teeth, the majority of them are performed in human single rooted tooth specimens with no evidence of caries or defects and radicular pathology. Considering tooth type, there is only one study performed in deciduous teeth [75]; the majority was achieved in permanent uniradicular human teeth. However, four studies used not only uniradicular but also multiradicular teeth [35, 73, 78, 82]. Besides, decayed teeth are also studied in deciduous [75] and permanent teeth [79].

Slaughterhouse bovine teeth [80] are convenient to use in antimicrobial PDT studies because of their match with human dentine; more precisely, their dentinal tubules are very similar to human teeth in quantity, size, diameter, morphology, and density. Moreover, bovine teeth [12] are simple to acquire and reduced size makes handling easier; in this term, they were used in 2 (7%) studies [72, 80]. Only one study, performed by Nagayoshi et al. [62], was executed in a resin block which attempts to mimic an* in vitro* model of apical periodontitis.

In the most common experimental model, dental specimens are decoronated to a standard length of 12 mm [67, 68, 78, 79] although gap value is very wide, from 8 [59, 77] to 15 mm [66, 81] or complete root canal length. Patency of apical foramina is established and then mechanical [35, 58, 61, 63–68, 70, 71, 73, 74, 76, 78, 79, 81, 82] instrumentation is performed using nickel-titanium rotary files, predominantly in a coronoapical (crown-down) technique [35, 58, 61, 63–68, 70, 71, 73, 74, 76, 78, 79, 81, 82] from canal orifice to apical third, until it reaches the value of master apical file (MAF) of K (Kerr) file 40 [58, 59, 68, 70]. However, other MAF have been described, such as 35 [57, 79, 81] and 30 [34].

In terms of irrigation with disinfecting agents, those are used for smear layer (SL) removal, lubrication, debris removal, and antimicrobial effects. SL is composed of organic and inorganic components like vital or necrotic pulp tissue, microorganisms, saliva, blood cells, and tooth structure. Among irrigation solutions, sodium hypochlorite (NaOCl) is the classical irrigant most used in endodontic therapy as a powerful antibacterial organic tissue dissolving agent.

NaOCl penetrates to a depth of approximately 130 *μ*m [92] to 160 *μ*m into dentinal tubules whereas tubular infection may occur closer to cementum-dentin junction (up to 1000 *μ*m) [93]. Bumb et al. [61] demonstrated in scanning electron microscope (SEM) penetration up to 1000 *μ*m into dentinal tubules of* E. faecalis* and compared penetrating power between a high power laser (Nd:YAG) that can go to a range of 400–850 *μ*m and PDT group that reaches as deep as 890–900 *μ*m.

Considering NaOCl as an unquestionable irrigation solution, its universal effective minimal concentration remains unclear. Apart from various outcomes reported by previous studies on comparative effectiveness of hypochlorite at different concentrations, it is regularly accepted that effectiveness of NaOCl is proportional to its concentration [24, 72, 94]. In antimicrobial PDT studies, NaOCl concentration range is between 0.5 [57, 67, 75, 80] and 6% [68, 78] and mainstream of studies used 2.5% NaOCl concentration [34, 58, 62, 64, 65, 70, 71, 74, 76, 79, 81]. Due to the fact that NaOCl has an influence upon only organic components of SL, it should be used with demineralizing agents, which can remove inorganic component of smear layer. Concerning SL elimination, only 3 readings [35, 70, 73] reported citric acid as a SL deletion, one at 10% [70] and two at 20% from the same author, Bonsor et al. [35, 73]. But the most popular SL removal is by far 17% ethylenediamine tetraacetic acid (EDTA) [34, 57–59, 61, 63, 65–69, 71, 74, 76–78, 81, 82].

### 3.7. Microorganisms

Reviewing literature on use of several microorganisms in PDT studies, authors could not evaluate* in vivo* studies in those terms, because no attempt was made to identify bacterial flora during culture process [35, 58, 73, 75] in four of six studies. Only Garcez et al., 2010 [74], and Jurič et al., 2014 [76], established microbiological identification.

Among all studies, we analysed 23 studies (all* in vitro* and* ex vivo*), and from those, 20 (87%) elected* Enterococcus faecalis* as substract to quantify antimicrobial PDT effectiveness.* E. faecalis* is a Gram-positive facultative anaerobe commonly detected in asymptomatic, persistent endodontic infections. Its prevalence in such infections ranges from 24% to 77% [95]. This finding can be explained by various survival and virulence factors [95] expressed by* E. faecalis*, including its ability to compete with other microorganisms, invade dentinal tubules, and resist nutritional deprivation.

*E. faecalis* was used not only in planktonic suspensions, but also in form of biofilms and the most common strain selected was ATCC29212. However, biofilm maturation time did not follow a linear pattern; besides, a huge discrepancy exists. Some authors used young biofilms with range of 2 [60, 68], 4 [60], and 7 days [81, 82] very distinct from mature biofilms performed with biofilms of 21 [61, 66], 28 [5, 69, 72, 77], and 70 days [59]. According to Kishen and Haapasalo 2010 [12], a mature biofilm is considered when maturation period is equal to or higher than 21 days and only 7 (30%) studies [5, 59, 61, 66, 69, 72, 77] respected this mature biofilm criteria. Apart from* E. faecalis*, other microorganisms were reviewed. Of note, in literature, the first PDT* in vitro* study was performed by Seal et al. 2002 [63] in root canals infected with* Streptococcus intermedius* (Gram-positive facultative anaerobe) biofilm with 2 days of maturation using TBO as PS and a helium-neon laser as light source.

## 4. Discussion

PDT, a technique with potentially significant antimicrobial properties, is a fairly recent approach in endodontic disinfection protocols. While the oral applications of PDT have been extensively tested, variations in study type and design limits the ability to synthesize or pool the available quantitative data, thereby permitting a formal meta-analysis and a systematic review.

Furthermore, many of the studies quantitatively measuring the degree of bacterial kill fail to report baseline bacterial counts or concentrations, thus limiting the ability to assess the bactericidal efficacy of PDT. Considering this apparent variation in reporting results among the studies analysed, it is difficult to provide a definitive assessment of the research question posed in this review. It is important to mention that PDT efficacy is shown in CFU or in percentage and logarithm (in form of log_10_); nonetheless, authors state this is pointless without the perception of the initial concentration. As an example, if we have an initial sample from a root canal of 10^7^ microorganisms and if after PDT approach we had 10^5^, statistically, 99% were killed, but there are still 100000 microorganisms left inside the root canal. Considering the variation in units at outcomes, the final results analysis is difficult.

Even though PDT has significant advantages (cited in Section 1), potential adverse events as tooth discoloration have been reported previously in root canal treatment when MB and TBO were used as PS [96]. It is also important that future clinical studies clearly report adverse events associated with PDT so that an estimation of the benefit-to-risk ratio from the use of PDT is feasible. Nonetheless, there were no adverse effects mentioned in the included studies of the current review.

PDT outcomes in literature have been reported by the dual combination of PS and a visible light source in the presence of oxygen; however, recently, Lins de Sousa et al. [97] analysed that twice-daily blue light of 420 nm, energy density of 72 Jcm^−2^, and irradiation time of 776 s without PS are a promising approach in the inhibition of five days'* Streptococcus mutans* matrix-rich biofilm development. It has remarkably inhibited the production of insoluble EPS, which is responsible for the scaffold of the extracellular biofilm matrix. The authors suggest that this evidence is very important to improve standardization in PDT procedures in the total absence of light as the evaluation of PS dark toxicity in some studies reviewed did not address this important issue.

In the literature, residual systemic photosensitization has also been reported as a potentially adverse event associated with the use of intravenous PS [98]; but this effect appears to not be associated with oral applications of PDT [99]. The role of PDT in root canal disinfection has been tested using several combinations of PS and light sources and has shown divergent results and these studies have revealed several limitations associated with antimicrobial PDT. For successful PDT to affect significant reduction or eradication of microorganisms, a PS is required which will show enough affinity for microorganisms without catalyzing photodamage to host tissues, a light source at a wavelength that can penetrate tissues (630–700 nm), and sufficient oxygenation to produce a level of reactive oxygen species (ROS) necessary to induce photodynamic lipid peroxidation and, as a consequence, necrosis and cell death. If there is photodamage to both tissues and microorganisms, efficacy will be suboptimal.

Microorganisms in the root canal flora and their growth mode were found to influence their susceptibility to PDT in a dose-dependent manner [100] and biofilms can be difficult to eradicate not only because of their effect as barriers to PS uptake, but also their ability to diffuse or attenuate light in the root canal dentinal tubules. Even dentin, dentin matrix, pulp tissue, bacterial lipopolysaccharides, and bovine serum albumin were found to significantly decrease PDT antimicrobial efficacy [101] and, as a consequence, an effort to enhance the PDT by nanoparticle-based technology appears promising [102]. Other strategies include the use of a PS solvent [103], efflux pump inhibitors [100], or photoactivated functionalized chitosan nanoparticles for disinfection and stabilization of the dentin matrix [104]. Because the application of PDT for additional reduction of the microbial load of root canal systems seems promising, it would be beneficial to identify the ideal combination of PS and light wavelength in preclinical studies and conduct future randomized controlled trials to test the effect of PDT on root canal disinfection in various indications.

## 5. Conclusion

PDT has been used thus far without a consensus-based, well-defined protocol, and therefore still remains at an experimental stage waiting for further optimization. Limited clinical information is currently available on the use of PDT in root canal disinfection. Currently, the level of evidence of available clinical studies to answer this question is low. Nevertheless, the results of this review suggest, based primarily on available* in vivo* studies, that PDT could perform well as an antimicrobial adjuvant. PDT appears to be a promising antimicrobial platform so further studies are warranted to optimize protocols using standardized laser and PS parameters to assess the PDT efficacy. Therefore, within the limits of the present review, one may conclude that the efficacy of PDT remains questionable, but promising. It is further suggested that an additional potential benefit from the use of PDT in root canal disinfection may exist where highly resistant bacteria are present in the root canal space, thus affecting the treatment prognosis. Further research is necessary to establish the appropriate PDT parameters allowing adequate antimicrobial action without harmful host side effects.

## Figures and Tables

**Table 1:** *In vitro* studies compilation.

Study type	Groups	% NaOCl	Substracte	Photosensitizer	Laser	Parameters evaluated	Conclusion
*In vitro, *16 studies							
Seal et al. 2002 [63]	*Test groups*: Group #1: PDT with 20 combinations of 4 TBO concentrations and 5 laser energy doses (*n* = 4). TBO (12.5, 25, 50, and 100 mg mL^−1^) incubated for 30 s. Laser (60, 90, 120, 300, and 600 s). Energy dose (2.1, 3.2, 4.2, 10.5, and 21 J). Group #2: NaOCl (*n* = 4). *Control group*: Light source: Canals (*n* = 4) were filled with reduced transport fluid (RTF) for 30 s followed by application of various laser light doses (60, 90, 120, 300, or 600 s). TBO only: Canals (*n* = 4) were filled with TBO at various concentrations (12.5, 25, 50, or 100 mg mL^−1^) and incubated for 30 s. No treatment: Canals (*n* = 17) were filled with RTF and incubated for 30 s.	3	*S. intermedius* (strain NS)	TBO [12.5, 25, 50, 100 *μ*g mL^−1^] Preincubation time (PIT): 30 s	Helium-neon [*λ*632.8 nm] Irradiation time (IT): 60 s, 90 s, 120 s, 300 s, and 600 s	Cell viability Colony-forming-unit – CFU (log_10_)	PDT is bactericidal to *S. intermedius* biofilms in root canals but is not as effective as irrigation with 3% NaOCl.
	Sample: 35 root canals from human uniradicular teeth						

Silva Garcez et al. 2006 [57]	*Test groups*: Group #1: L^−^AZ^−^ Group #2: L^+^AZ^−^ Group #3: L^−^AZ^+^ Group #4: L^+^AZ^+^ Group #5: NaOCl *Control group*: Canals filled with BHI broth and incubated for 24 h.	0.5	*Enterococcus faecalis *(ATCC1494)	AZ paste [0.01%] PIT: 300 s Paste composition: urea peroxide 10%, detergent 15% (Tween 80) and vehicle 75% (carbowax).	GaAlAs diode [*λ*685 nm] IT: 180 s	Cell viability CFU (log_10_)	In root canals, PDT showed 99.2% *E. faecalis* reduction, whereas 0.5% NaOCl achieved 93.25%.
	Sample: 30 root canals from human uniradicular teeth (upper central incisors and upper canines)						

Garcez et al. 2007 [34]	*Test groups*: Group #1: PDT Group #2: RCT (root canal treatment) with NaOCl Group #3: Combined treatment (PDT + ET with NaOCl) *Control group*: Teeth with 3-day biofilms + BHI for 24 h	2.5	*Proteus mirabilis * (XEN44) *Pseudomonas aeruginosa * (XEN5)	PEI/e6 [NS] PIT: 600 s	MMOptics [*λ*660 nm] IT: NS	Bioluminescence imaging Cell viability CFU (log_10_)	NaOCl reduced bacteria by 90% while PDT alone reduced bioluminescence by 95%.
	Sample: 10 root canals from uniradicular human teeth (upper central incisors and upper canines)						

George and Kishen 2008 [59, 103] *In vitro* *Ex vivo*	*Test groups*: Group #1: RCT with NaOCl Group #2: PDT Group #3: RCT + PDT in an emulsion of H_2_O_2_ : triton-X100 in the ratio 75 : 24.5 : 0.5 Group #4: RCT + an emulsion of H_2_O_2_ : triton-X100 in the ratio 75 : 24.5 : 0.5 *Control group*: Root canal not subject to any treatment	IV: 1 EV: 5.2	*E. faecalis* (strain NS)	MB [1, 5, 10, 15, 20, 25 *μ*M] PIT: 600 s (in the dark) Dark toxicity was evaluated Perfluorodecahydronaphthalene (oxygen carrier) H_2_O_2_(oxider) Triton-X100 (nonionic detergent)	Power Technology Inc. [*λ*664 nm] IT: NS	CSLM Photooxidation activity Singlet oxygen generation Cell viability CFU (log_10_)	NaOCl showed no viable bacteria after 4 h, but 60% of the root canal shavings confirmed bacterial growth after 24 h. PDT alone or + NaOCl showed the absence of bacteria even after 24 h.
	Sample: *in vitro*: *E. faecalis* biofilms grown on a glass coverslip that was fixed covering a grove (6 mm diameter) made at the bottom part of a Petri dish						
	*Ex vivo *(16–24 years)*: *30 root canals from human uniradicular teeth (anterior teeth)						

Meire et al. 2009 [64] *In vitro* *Ex vivo*	*Test groups*: Group #1: Nd:YAG laser (*n* = 10) Group #2: KTP laser (*n* = 10) Group #3: PDT (*n* = 10) Group #4: NaOCl (*n* = 10) *Control group*: Group #5: teeth with no treatment (*n* = 20) – positive control Group #6: uninoculated teeth (*n* = 3) – negative control	2.5	*E. faecalis * (ATCC10541)	TBO [12.7 mg mL^−1^] PIT: 120 s	Denfotex [*λ*635 nm] IT: 150 s	Cell viability CFU (log_10_) Solid phase cytometry Epifluorescence microscopy	PDT was less effective than NaOCl (15 min) in reducing *E. faecalis*, both in aqueous suspension and in the infected tooth model.
	Sample: 60 uniradicular human teeth						

Souza et al.2010 [65]	*Test groups*: Group #1: PDT with MB + NaOCl (*n* = 16) Group #2: PDT with TBO + NaOCl (*n* = 10) Group #3: PDT with MB + NaCl (*n* = 16) Group #4: PDT with TBO + NaCl (*n* = 16) *Control groups*: *⌀*	2.5	*E. faecalis* (MB35)	MB/TBO [15/15 *μ*g mL^−1^] PIT: 120 s	MMOptics [*λ*660 nm] IT: 240 s	SEM Cell viability CFU (log_10_)	PDT did not significantly enhance disinfection after chemomechanical preparation using NaOCl as irrigant.
	Sample: 70 uniradicular human teeth						

Nagayoshi et al. 2011 [62]	*Test groups*: Group #1: 5 W, 30 s, PS (+) Group #2: 5 W, 60 s, PS (+) Group #3: 5 W, 120 s, PS (+) Group #4: 5 W, 120 s, PS (−) *Control groups*: Group #5: NaCL: negative control Group #6: NaOCl: positive control	2.5	*E. faecalis* (ATCC29212)	Indocyanine green [12. mg mL^−1^] PIT: 60 s	P-Laser [*λ*805 nm] IT: 30, 60, 120 s	Cell viability CFU (log_10_) Temperature	PDT had nearly the same antimicrobial effect as 2.5% NaOCl.
	Sample: *in vitro* model of apical periodontitis in resin blocks						

Nunes et al. 2011 [66]	*Test groups*: Group #1: OF/IT90 (*n* = 10) Group #2: OF/IT180 (*n* = 10) Group #3: NOF/IT90 (*n* = 10) Group #4: NOF/IT180 (*n* = 10) *Control groups*: Group #5: untreated (*n* = 10) Group #6: NaOCl: positive control (*n* = 10)	1	*E. faecalis* (ATCC29212)	MB [100 *μ*g mL^−1^] PIT: 300 s	Thera Lase [*λ*660 nm] IT: 90, 180 s	Cell viability CFU (log_10_)	The highest percentage of *E. faecalis* reduction was achieved with NaOCl. The use of intracanal fiber during PDT does not reveal improvement.
	Sample: 60 uniradicular human teeth						

Poggio et al. 2011 [67]	*Test groups*: Group #1: PDT (*n* = 10) Group #2: PDT + NaOCl (*n* = 10) Group #3: TBO (*n* = 10) Group #4: PDT (*n* = 10) – more time than in group 1 *Control groups*: Group #5: NaOCl: positive control (*n* = 10)	0.5 5	*Streptococcus mutans *(CCUG35176) *E. faecalis *(ATCC19433) *Streptococcus sanguis *(CCUG17826)	TBO [100 *μ*g mL^−1^] PIT: 60 s	FotoSan [*λ*628 nm] IT: 30, 60 s	Cell viability	*In vitro *antimicrobial efficacy of 5% NaOCl is higher than PDT.
	Sample: 100 root canals from human uniradicular teeth						

Rios et al. 2011 [68]	*Test groups*: Group #1: NaOCl Group #2: TBO Group #3: Light Group #4: PDT Group #5: PDT + NaOCl *Control groups*: The experimental conditions were repeated seven independent times with 15 total experimental samples. Both negative (no growth) and positive (growth without any treatment) controls were done for each independent experiment.	6	*E. faecalis* (OG1X) A derivative of an oral isolate that has been shown to be cariogenic	TBO [NS] PIT: 30 s	FotoSan [*λ*628 nm] IT: 30 s	SEM Cell viability CFU (log_10_)	The bacterial survival rate of the NaOCl/PDT group (0.1%) was significantly lower than the NaOCl (0.66%) and PDT groups (2.9%).
	Sample: uniradicular human teeth (total number of teeth unknown)						

Cheng et al. 2012 [69]	*Test groups*: Group #1: Nd:YAG Group #2: Er:YAG/NaOCl/NS/DW Group #3: Er:YAG/NS/DW Group #4: Er,Cr:YSGG Group #5: PDT *Control groups*: Group #6: NaOCl: positive control Group #7: normal saline: negative control	5.25	*E. faecalis* (ATCC4083)	MB [50 *μ*g mL^−1^] PIT: 60 s	Nd:YAG [*λ*1064 nm] IT: 16 s Er:YAG [*λ*2940 nm] IT: 20 s Er,Cr:YSGG [*λ*2780 nm] IT: 4 s Lit-601 [*λ*660 nm] IT: 60 s	SEM Cell viability CFU (log_10_)	PDT was less effective than NaOCl at surface of the root and 100, 200, and 300 *μ*m inside the dentinal tubule.
	Sample: 220 uniradicular human teeth						

Vaziri et al. 2012 [70]	*Test groups*: Group #1: NaOCl (*n* = 15) Group #2: Laser + NaOCl (*n* = 15) Group #3: PDT (*n* = 15) Group #4: PDT + NaOCl (*n* = 15) Group #5: chlorhexidine (*n* = 15) *Control groups*: Group #6: no treatment: positive control Group #7: without inoculation of bacterium: negative control	2.5	*E. faecalis* (ATCC29212)	TBO [15 *μ*g mL^−1^] PIT: 300 s	FotoSan [*λ*625 nm] IT: 60 s	Cell viability CFU (log_10_)	NaOCl showed better results than PDT. However, PDT + NaOCl showed the best result.
	Sample: 90 root canals from 90 uniradicular human teeth						

Pileggi et al. 2013 [60]	*Test groups*: Group #1: PDT (Eosin-Y) with Light+ and L− Group #2: PDT (Rose bengal) with Light+ and L− Group #3: PDT (Curcumin) with Light+ and L− *Control groups*: Group #4: NaOCl positive control	3	*E. faecalis* (135737)	Eosyn-Y/RB/curcumin [50 *μ*g mL^−1^] PIT: 1800 s	Optilux 501 [*λ*380–500 nm] IT: 240 s	Cell viability CFU (log_10_)	In BS, PDT significantly reduced *E. faecalis *viability. For biofilm, PDT completely suppressed *E. faecalis*.
	Sample: *E. faecalis* 135737 culture collection of the University Hospitals of Geneva; CH was used for the inactivation assays because of its prominent role in endodontic infections						

Bumb et al. 2014 [61]	*Test groups:*Group #1: PDT (MB) with Light+ *Control groups:* Group #2: no treatment (*n* = 10) Positive control	3	*E. faecalis* (ATCC29212)	MB [25 mg mL^−1^] PIT: 600 s	Diode laser [*λ*910 nm] IT: NS	SEM Cell viability CFU (log_10_)	Bacterial reduction in PDT group was 96.70%. PDT potential to be used as an adjunctive antimicrobial procedure.
	Sample: 20 uniradicular human teeth						

Gergova et al. 2015 [71]	*Test groups:*Group #1: lasers (*n* = 40) #1.1: Nd:YAG (*n* = 20) #1.2: diode (*n* = 20) Group #2: PDT (*n* = 60) #2.1: FotoSan (*n* = 20) #2.2: without laser – dark control (*n* = 20) #2.3: without PS – light control (*n* = 20) Group #3: iontophoresis (*n* = 120) #3.1: Cupral (*n* = 40) #3.2: Ca(OH)_2_ (*n* = 40) #3.3: I_2_/KI_2_ (*n* = 40) Group #4 (*n* = 60) #4.1: 2% Chx (*n* = 20) #4.2: 2.5% NaOCl (*n* = 20) #4.3: 30% H_2_O_2_ (*n* = 20) *Control groups:* Group #5: PBS (*n* = 20) Positive control	2.5	Two control strains from the American Type Culture Collection (ATCC): Methicillin sensitive* Staphylococcus aureus* (ATCC29213) *E. faecalis* (ATCC29212) Clinical isolates served as multidrug-resistant: *S. pyogenes* *S. intermedius* *E. coli K. pneumonia* *E. cloacae* *S. marcescens* *M. morganii* *P. aeruginosa* *A. baumannii* *C. albicans*	TBO [15 *μ*g mL^−1^] PIT: NS	FotoSan [*λ*625 nm] IT: 300 s	SEM Cell viability CFU (log_10_) X-ray laser particle sizer	2.5% NaOCl is the most satisfactory result; however, PDT with FotoSan, H_2_O_2_, and all tested types of iontophoresis all showed strong disinfection potential without statistical significance.
	Sample: 300 uniradicular human teeth						

Wang et al. 2015 [72]	*Test groups:*Group #1: PDT (*n* = 10) Group #2: ultrasonic irrigation + NaOCl #2.1: US + 0.5% NaOCl (*n* = 10) #2.2: US + 1% NaOCl (*n* = 10) #2.3: US + 2% NaOCl (*n* = 10) #2.4: US + 2.5% NaOCl (*n* = 10) #2.5: US + 5.25% NaOCl (*n* = 10) Group #3: ultrasonic irrigation + PDT + NaOCl #3.1: US + PDT + 0.5% NaOCl (*n* = 10) #3.2: US + PDT + 1% NaOCl (*n* = 10) #3.3: US + PDT + 2% NaOCl (*n* = 10) #3.4: US + PDT + 2.5% NaOCl (*n* = 10) #3.5: US + PDT + 5.25% NaOCl (*n* = 10) *Control groups:* Group #4: ultrasonic irrigation with 0.9% NaCl (*n* = 10) Negative control	0.5 1 2 2.5 5.25	*E. faecalis *(ATCC33186)	MB [100 *μ*M] PIT: 600 s	Diode laser [*λ*670 nm] IT: 300 s	SEM Cell viability CFU (log_10_)	PDT alone is less efficient than even the 0.5% NaOCl ultrasonic irrigation under the condition of this experiment.
	Sample: 120 intact bovine incisors						

**Table 2:** *In vivo* studies collection.

Study type	Groups	% NaOCl	Substracte	Photosensitizer	Laser	Parameters evaluated	Conclusion
*In vivo, *6 studies							
Bonsor et al. 2006 [35, 73]. Private general dental practice in Scotland by the same operator, UK.	Group #1 (73% molars): Three samples (*n* = 32): (1.1) After gaining access to the root canal. (1.2) After apex location and PDT process carried out. (1.3) After completion of the canal preparation using citric acid and NaOCl. Group #2 (76% molars): Three samples (*n* = 32): (2.1) After gaining access to the root canal. (2.2) After conventional preparation using 20% citric acid and NaOCl. (2.3) After a subsequent PDT. *Control groups*: ⌀ Random allocation? Yes	2.25	Human dentine of the canal's walls. No attempt was made to identify the specific bacterial flora during the culturing process.	TBO [12.7 mg L^−1^] PIT: 60 s	SaveDent Diode laser [*λ*633 ± 2 nm] IT: 120 s	Scores for levels of infection	PDT showed best results (93%) when compared to conventional irrigants solutions like NaOCl and acid citric (76%).
	Sample (16–70 years): 64 root canals with closed apices randomly selected from uni- and multiradicular teeth of 14 healthier patients presented with symptoms of irreversible pulpitis or periradicular periodontitis						

Bonsor et al. 2006 [35, 73]. Private general dental practice in Scotland by the same operator, UK.	Group #1: Three samples (*n* = 30) (1.1) After gaining access to the root canal. (1.2) After conventional endodontic therapy with NaOCl (1.3) After PDT. *Control groups*: ⌀ Random allocation? Yes	2.25	Human dentine of the canal's walls. No attempt was made to identify the specific bacterial flora during the culturing process.	TBO [NS] PIT: 60 s	SaveDent Diode laser [*λ*633 ± 2 nm] IT: 60, 120 s	Scores for levels of infection	PDT showed best results when compared to conventional irrigant solutions.
	Sample (16–70 years): 64 root canals with closed apices randomly selected from uni- and multiradicular teeth of 14 healthier patients presented with symptoms of irreversible pulpitis or periradicular periodontitis						

Garcez et al. 2008 [58]. Private dental practice in São Paulo by the same operator, Brazil.	Group #1: Three samples (*n* = 30) (1.1) After gaining access to the root canal. (1.2) After conventional endodontic therapy with NaOCl. (1.3) After PDT. Group #2: Two samples after 1 week with Ca(OH)_2_. (2.1) After 2nd conventional endodontic therapy with NaOCl. (2.2) After 2nd PDT. *Control groups*: ⌀ Random allocation? Yes	2.5	Human dentine of the canal's walls. No attempt was made to identify the specific bacterial flora during the culturing process.	PEI/e6 [60 *μ*mol L^−1^] PIT: 120 s	MMOptics Diode laser [*λ*660 nm] IT: 240 s	Cell viability CFU (log_10_)	The use of PDT leads to a significant further reduction of bacterial load, and a second appointment PDT is even more effective than the first.
	Sample **(**21–35 years): 20 selected cases of patients presenting with symptoms of irreversible pulpitis or periradicular periodontitis in anterior teeth (incisors and canines) selected at random						

Garcez et al. 2010 [74]. Private dental practice in São Paulo by the same operator, Brazil.	Group #1: Three samples (*n* = 30) (1.1) After gaining access to the root canal. (1.2) After conventional endodontic therapy with NaOCl. (1.3) After PDT. *Control groups*: ⌀ Random allocation? No	2.5	Biofilms At least 1 microorganism resistant to antibiotic medication.	PEI/e6 [≈19 *μ*g mL^−1^] PIT: 120 s	MMOptics Diode laser [*λ*660 nm] IT: 240 s	Microbiological identification Antibiogram analyses Cell viability CFU (log_10_)	NaOCl reduced to 0.8 species per root canal. After PDT, microorganism growth was not detected on any of the samples.
	Sample (17–52 years): 30 anterior uniradicular human teeth with previous endodontic treatment from 21 patients without random allocation						

Prabhakar et al. 2013 [75]. Department of Pedodontics and Preventive Dentistry, Bapuji Dental College and Hospital, Davangere, Karnataka, India.	Group #1: Three samples (*n* = 12) (1.1) After gaining access to the root canal. (1.2) After conventional endodontic therapy with NaOCl (1.3) After PDT. *Control groups*: ⌀ Random allocation? No	0.5	Culture samples	MB [50 *μ*g mL^−1^] PIT: 300 s	Silberbauer low level laser Diode laser [*λ*660 nm] IT: NS	Cell viability CFU (log_10_)	PDT showed best results than NaOCl.
	Sample (4–7 years): 12 human deciduous molars with caries lesions affecting the pulp and diagnosed as necrotic pulps (pulpectomies) from twelve children without random allocation						

Jurič et al. 2014 [76]	Group #1: Three samples (*n* = 21) (1.1) After gaining access to the root canal (initial) (1.2) After chemomechanical preparation (1.3) After chemomechanical preparation + PDT *Control groups*: ⌀ Random allocation? Yes	2.5	Biofilms	Helbo blue PS [10 mg mL^−1^] PIT: 120 s Phenothiazinium chloride	Helbo system Diode laser [*λ*660 nm] IT: 60 s	Microbiological identification Cell viability CFU (log_10_)	Although endodontic re-treatment (ERT) alone produced a significant reduction in the number of bacteria species, the combination of ERT + PDT was statistically more effective.
	Sample (20–45 years): 21 anterior uniradicular human teeth (incisors or canines) with previous endodontic treatment from 21 patients with random allocation						

**Table 3:** *Ex vivo* studies compilation.

Study type	Groups	% NaOCl	Substracte	Photosensitizer	Laser	Parameters evaluated	Conclusion
*Ex vivo*, 7 studies							
Lim et al. 2009 [77]	*Experiment #1* *Test groups:*Group #1: laser (*n* = 10) Group #2: PDT + PS in water (*n* = 10) Group #3: NaOCl (*n* = 10) Group #4: PDT + PS in Mix (*n* = 10) *Control groups:*Group #5: no treatment (*n* = 15): positive control *Experiment #2* *Test groups:* Group #1: PDT + PS in water (*n* = 6) Group #2: PDT + PS in Mix (*n* = 6) Group #3: cleaning and shaping (*n* = 6) Group #4: PDT + PS in Mix + cleaning and shaping (*n* = 6) *Control groups:* Group #5: no treatment (*n* = 6): positive control	5.25	*E. faecalis* (ATCC29212)	MB [100 *μ*M] PIT: NS Dissolved in water and MIX	Model PPM35 [*λ*660 nm] IT: 1200 s	Cell viability CFU (log_10_)	NaOCl showed best results that conventional PDT.
	Sample: 85 freshly extracted uniradicular human teeth						

Ng et al. 2011 [78]	*Test groups:*Group #1: chemomechanical debridement with NaOCl (*n* = 26) Group #2: PDT + chemomechanical debridement with NaOCl (*n* = 26) *Control groups:* ⌀	6	Human intracanal dentinal shavings	MB [50 *μ*g mL^−1^] PIT: 300 s	BWTEK Inc. [*λ*665 nm] IT: 150 s-break 150 s-150 s	DNA probes Cell viability CFU (log_10_)	PDT + NaOCl showed better results when compared to NaOCl alone.
	Sample: 52 freshly extracted human teeth with pulpal necrosis (9 incisors, 5 canines, 12 premolars, and 26 molars)						

Stojicic et al. 2013 [5]	*Test groups:*Group #1: 0.1% EDTA + 0.1% H_2_O_2_ (1 min) Group #2: 0.1% EDTA + 0.1% Chx (1 min) Group #3: MB 15 (PIT = 5 min) 1 min LASER Group #4: MB 100 (no PIT) 1 min LASER Group #5: MB 100 (PIT = 5 min) 1 min LASER Group #6: MB 100 (PIT = 5 min) + 0.1% EDTA + 0.1% H_2_O_2_ 1 min LASER Group #7: MB 100 (PIT = 5 min) + 0.1% EDTA + 0.1% Chx 1 min LASER Group #8: 2% CHX 1 min Group #9: 1% NaOCl 1 min Group #10: 2% NaOCl 1 min *Control groups:* Group #11: 1 mL of sterile water for 6 min: positive control	1.0 2.0	*E. faecalis* (VP3-181, VP3-180, Gel 31, and Gel 32)	MB BS – [15 *μ*mol L^−1^] Biofilm – [100 *μ*mol L^−1^] PIT: 300 s	Twin Laser (MMOptics) [*λ*660 nm] IT: 30 s, 60 s, 180 s	Viability staining CLSM	Modified PDT killed 20 times more than conventional PDT and up to 8 times more than 2% CHX and 1% NaOCl.
	Sample: Bacterial plaque from 3 adult volunteers used in 4 strains of *E. faecalis* (originally isolated from root canals of the teeth with periapical lesions)						

Bago et al. 2013 [79]	*Test groups:*Group #1: NaOCl (*n* = 20) Group #2: EndoActivator + NaOCl (*n* = 20) Group #3: Diode laser (*n* = 20) Group #4: PDT (*n* = 20) Group #5: PDT + 3D Endoprobe (*n* = 20) *Control groups:* Group #6: NaCl (*n* = 10): positive control	2.5	*E. faecalis* (ATCC29212)	Phenothiazine chloride/TBO [10 mg mL^−1^]/[155 *μ*g mL^−1^] PIT: 60, 120 s	Helbo and Laser HF [*λ*660 nm] IT: 60 s The 2 lasers have the same wavelength.	SEM Cell viability CFU (log_10_) PCR	PDT using both laser systems and the sonic activated NaOCl irrigation were significantly more effective than diode irradiation and single NaOCl.
	Sample: 120 uniradicular human teeth (mandibular incisors and maxillary second premolar extracted because of periodontal disease or extensive carious lesions without root caries or previous endodontic treatment)						

Hecker et al. 2013 [80]	*Test groups:*Group #1: NaOCl (0.5%, 1.0% or 3.0%) for 30, 60, or 600 s (*n* = 10) Group #2: NaOCl (0.5%, 1.0% or 3.0%) for 30, 60, or 600 s + neutralizing solution (*n* = 10) Group #3: PDT (*n* = 10) *Control groups* Group #4: TBO (only) (*n* = 10) Group #5: laser (only) (*n* = 10) Group #6: apical section as sterile control: negative control Group #7: middle section to confirm successful infection: positive control	0.5 1.0 3.0	*E. faecalis *(ATCC29212)	TBO [NS] PIT: 60 s	Pact 200 system [*λ*635 nm] IT: 240, 360 s	Cell viability CFU (log_10_) SEM	The antibacterial PDT system did not achieve sufficient disinfection when compared to NaOCl.
	Sample: roots of freshly extracted permanent bovine mandibular incisors (total number of teeth unknown)						

Muhammad et al. 2014 [82]	*Test groups:*Group #1: PDT with Aseptim Plus - LED disinfection system (*n* = 10) Group #2: PDT with diode laser Group #3: PUI + 17% EDTA + 2.6% NaOCl *Control groups* Group #4: no inoculation (*n* = 2) negative control Group #5: with inoculation (*n* = 2): positive control	2.6	*E. faecalis* *S. salivarius* (ATCC7073) *P. gingivalis* (ATCC 33277) *P. intermedia*	TBO [15 *μ*g mL^−1^] PIT: 60 s	LED [*λ*635 nm] Diode laser [*λ*650 nm] IT_(LED/DIODE)_: 120 s	SEM Scores for levels of infection (Bonsor et al. 2006 [35, 73])	The group treated with PUI + 2.5% NaOCl + 17% EDTA solution has the best results when compared to PDT with 2 different light sources.
	Sample: 30 roots obtained from 50 extracted human single and multirooted teeth						

Xhevdet et al. 2014 [81]	*Experiment #1* *E. faecalis* (*n* = 78) *Test groups:*Group #1: PDT (1 min) (*n* = 13) Group #2: PDT (3 min) (*n* = 13) Group #3: PDT (5 min) (*n* = 13) Group #4: NaOCl + PBS (*n* = 13) Group #5: NaOCl + 10 sec passive ultrasonic irrigation (PUI) (*n* = 13) *Control groups:* Group #6: no treatment (*n* = 13): positive control *Experiment #2* *C. albicans* (*n* = 78) *Test groups:* Group #1: PDT (1 min) (*n* = 13) Group #2: PDT (3 min) (*n* = 13) Group #3: PDT (5 min) (*n* = 13) Group #4: NaOCl + PBS (*n* = 13) Group #5: NaOCl + 10 sec PUI (*n* = 13) *Control groups:* Group #6: no treatment (*n* = 13): positive control	2.5	*E. faecalis* (ATC29121) *Candida albicans * (ATCC60193)	Phenothiazine chloride [10 mg mL^−1^] PIT: 60 s	HELBO [*λ*660 nm] IT: 60, 180, 300 s	Flow cytometry SEM Cell viability CFU (log_10_)	Irrigation with NaOCl showed similar results to 5 min irradiation of PDT.
	Sample: 156 extracted uniradicular human teeth						

**Table 4:** PDT microbial reduction outcomes.

Author	Study type	Microorganisms	Efficacy (% or log_10_)
Seal et al. 2002 [63]	*In vitro*	*S. intermedius*	5 log_10_
Bonsor et al. 2006 [35, 73]	*In vivo*	Polymicrobial infected teeth	96.7
Bonsor et al. 2006 [35, 73]	*In vivo*	Polymicrobial infected teeth	91
Silva Garcez et al. 2006 [57]	*In vitro*	*E. faecalis*	99.2
Garcez et al. 2007 [34]	*In vitro*	*P. mirabilis *and* P. aeruginosa *	98
Garcez et al. 2008 [58]	*In vivo*	Polymicrobial human dentine of the canal's walls	99.9
George and Kishen 2008 [59, 103]	*In vitro/ex vivo*	*E. faecalis*	100
Lim et al. 2009 [77]	*Ex vivo*	*E. faecalis*	99.99
Meire et al. 2009 [64]	*In vitro/ex vivo*	*E. faecalis *	1–1.5 log_10_
Souza et al. 2010 [65]	*In vitro*	*E. faecalis*	99.48
Garcez et al. 2010 [74]	*In vivo*	Polymicrobial infected teeth	100
Nagayoshi et al. 2011 [62]	*In vitro*	*E. faecalis*	99.99
Ng et al. 2011 [78]	*Ex vivo*	Human intracanal dentinal shavings	70
Nunes et al. 2011 [66]	*In vitro*	*E. faecalis*	99.41
Poggio et al. 2011 [67]	*In vitro*	*S. mutans; E. faecalis*, and *S. sanguis *	91.49
Rios et al. 2011 [68]	*In vitro*	*E. faecalis *	99.9
Bago et al. 2013 [79]	*Ex vivo*	*E. faecalis*	99.99
Cheng et al. 2012 [69]	*In vitro*	*E. faecalis*	96.96
Pileggi et al. 2013 [60]	*In vitro*	*E. faecalis*	96.7
Stojicic et al. 2013 [5]	*Ex vivo*	*E. faecalis*	100
Vaziri et al. 2012 [70]	*In vitro*	*E. faecalis*	82.3%
Hecker et al. 2013 [80]	*Ex vivo*	*E. faecalis*	Not specified
Prabhakar et al. 2013 [75]	*In vivo*	Polymicrobial infected teeth	99.99
Bumb et al. 2014 [61]	*In vitro*	*E. faecalis*	96.7
Gergova et al. 2015 [71]	*In vitro*	*S. aureus; E. faecalis*; *S. pyogenes; S. intermedius; E. coli; K. pneumonia; E. cloacae; S. marcescens; M. morganii; P. aeruginosa*; *A. baumannii; C. albicans*	42–54
Jurič et al. 2014 [76]	*In vivo*	Polymicrobial infected teeth	100
Muhammad et al. 2014 [82]	*Ex vivo*	*E. faecalis*; *S. salivarius*; *P. gingivalis; P. intermedia*	Not specified
Xhevdet et al. 2014 [81]	*Ex vivo*	*E. faecalis *and *C. albicans*	71.59
Wang et al. 2015 [72]	*In vitro*	*E. faecalis*	5.20 log_10_

**Table 5:** Studies compilation: laser, photosensitizer, and fiber applied.

Study type	Year	Author	Laser					Photosensitizer		PDT outcomes	
			Wavelength (nm)	Diameter of fiber (*μ*m) Working length (WL) EL	Power of output (mW)	Power of density (mW/cm^2^)	Energy fluence (J/cm^2^)	Type	Concentration (*μ*g/mL)	+	−
*In vitro*, 16 studies	2002	Seal et al. [63]	632.8	*Without fiber* Light at the orifice of the access cavity	35	—	42.9, 63.3, 85.7, 214.3, 428.6	TBO	12.5, 25, 50, 100		−
	2006	Silva Garcez et al. [57]	685	365 WL-1 mm Helicoidal movements, from apical to cervical	50	—	—	AZpaste	0.01% AZ paste	+	
	2007	Garcez et al. [34]	660	200 WL Spiral movements, from apical to cervical	40	—	5, 10, 20 e 40	PEI/e6	NS	+	
	2008	George and Kishen [59, 103]	664	400 NS NS	30	—	63.69	MB	1, 5, 10, 15, 20, 25 μM	+	
	2009	Meire et al. [64]	635	400 WL IV: static spherical tip in the centre of the liquid EV: 70% of the light radially as a cylinder uniformly and 30% at the tip; moved up and down in the canal	100	—	—	TBO	12.7 mg mL^−1^		−
		Souza et al. [65]	660	300 NS Spiral movements from apical to cervical	40	—	—	MB/TBO	15/15		−
	2011	Nagayoshi et al. [62]	805	400 WL NS	5000	—	—	Indocyanine green	12. mg mL^−1^	≈	
	2011	Nunes et al. [66] Study with and without fiber	660	*Without fiber* Handpiece placed in root canal orifice 216 WL Spiral movements from apical to cervical	90	300	—	MB	100		−
		Poggio et al. [67]	628	500 WL-3 mm Endotip guide to the apical parts	—	—	—	TBO	100		−
		Rios et al. [68]	628	NS WL-2/3 mm NS	—	—	—	TBO	NS		−
	2012	Cheng et al. [69]	*Nd:YAG* [*λ*1064 nm]	*Nd:YAG* 200 WL-1 Spiral movement	—	—	—	MB	50		−
			*Er:YAG* [*λ*2940 nm]	*Er:YAG* 300 Orifice of root canal NS							
			*Er,Cr:YSGG* [*λ*2780 nm]	*Er,Cr:YSGG* 415 WL-1 NS							
			*Diode* [*λ*660 nm]	*Diode* 2000 WL-3 mm NS							
		Vaziri et al. [70]	625	*Without fiber*	—	200	12	TBO	15		−
		Pileggi et al. [60]	380–500	10.4 mm Light source 20 mm away from the bacteria NS	—	450	108	Eosin-Y RB Curcumin	[*BS*] All 1 *μ*M *Biofilms* Eosin-Y 100 *μ*M RB/curcumin 10 *μ*M	+	
	2014	Bumb et al. [61]	910	NS WL-2 mm Circular movements, from apical to cervical	1000	—	—	MB	25 mg mL^−1^	+	
	2015	Gergova et al. [71]	660	200 WL Helicoidal traction movements, from apical to cervical	100	—	—	TBO	0.1 mg mL^−1^		−
	2015	Wang et al. [72]	670	400 NS NS	50	—	—	MB	60 *μ*M		−

*In vivo*, 6 studies	2006	Bonsor et al. [35, 73]	633	Flexible hollow tube WL-4 mm Moved up and down about 3 mm at 20 s	100	—	—	TBO	12.7 mg mL^−1^	+	
		Bonsor et al. [35, 73]	633	Flexible emitter tip WL-4 mm Moved up and down about 3 mm at 20 s	100	—	—	TBO	NS	+	
	2008	Garcez et al. [58]	660	300 WL Spiral movements, from apical to cervical	40	—	—	PEI/e6	60 *μ*mol L^−1^	+	
	2010	Garcez et al. [74]	660	200 WL-1 mm Spiral movements	40	—	—	PEI/e6	≈19	+	
	2013	Prabhakar et al. [75] Deciduous teeth	660	*Without fiber*	30		8.6	MB	50	+	
	2014	Jurič et al. [76]	660	450 WL Static	100	—	—	Phenothiazinium chloride		+	

*Ex vivo*, 7 studies	2009	Lim et al. [77]	660	400 Root canal orifice Static	30	—	—	MB	100 *μ*M		−
	2011	Ng et al. [78]	665	250 10 mm 360°	—	100	30	MB	50 *μ*g mL^−1^	+	
		Stojicic et al. [5]	660	NS [*BS*] Long optical fiber with a diameter 0.4 mm *Biofilm* Conical frustum tip with the end diameter of 5 mm NS	40	—	—	MB	[BS] 15 *μ*mol L^−1^ Biofilm 100 *μ*mol L^−1^	+	
		Bago et al. [79]	660	320 WL Spiral movements, from apical to cervical	100	—	—	Phenothiazine chloride/TBO	10 mg mL^−1^/155	+	
	2013	Hecker et al. [80]	635	*Without fiber*	200	—	—	TBO	NS		−
	2014	Muhammad et al. [82]	650	300 WL Moved all along the canals	20	—	—	TBO	15 *μ*g mL^−1^		−
		Xhevdet et al. [81]	660	NS WL Light at the tip and from the lateral sides	100	100	—	Phenothiazine chloride	10 mg mL^−1^	≈	
